# The Role of Leukoaraiosis and Microbleeds in Acute Ischemic Stroke Outcome Prediction

**DOI:** 10.3390/jcm15051879

**Published:** 2026-03-01

**Authors:** Aleksandra Aracki-Trenkic, Dunja Radovanović, Bruno Law-ye, Didier Dormont, Nadya Pyatigorskaya, Milica Živanović

**Affiliations:** 1 Department of Radiology, Clinical Center Nis, Boulevard Dr Zorana Djindjica, 48, 18000 Nis, Serbia; radovanoviczdunja@gmail.com (D.R.); milicazivanovic2601@gmail.com (M.Ž.); 2 Faculty of Medicine, University of Nis, Boulevard Dr Zorana Djindjica 81, 18000 Nis, Serbia; 3 Department of Neuroradiology, University Hospital Pitié-Salpêtrière, APHP, 47 Boulevard de l’Hopital, 75651 Paris, CEDEX 13, France; brunolawye@hotmail.fr (B.L.-y.); didier.dormont@psl.aphp.fr (D.D.); nadya.pyatigorskaya@aphp.fr (N.P.); 4 Pierre and Marie Curie Faculty of Medicine, Sorbonnes Universités, UPMC Univ Paris 06, UMR S 1127, CNRS UMR 7225, 75013 Paris, France

**Keywords:** acute ischemic stroke, MRI, leukoaraiosis, cerebral microbleeds, aCBF, functional outcome

## Abstract

**Background/Objectives**: Acute ischemic stroke (AIS) is one of the leading causes of mortality worldwide and the primary cause of acquired neurological disability in adults. As part of a stroke magnetic resonance (MR) protocol, fluid-attenuated inversion recovery (FLAIR) plays an important role in the detection and assessment of the degree of leukoaraiosis (LA), while susceptibility-weighted angiography (SWAN) detects cerebral microbleeds (CMBs). The present study sought to examine the association of the degree of LA and CMBs with absolute cerebral blood flow (aCBF) values and functional outcome prediction in patients with AIS. **Methods**: We conducted a cross-sectional study including a total of 205 male and female patients. All of the patients underwent brain magnetic resonance imaging (MRI) examinations in the first 24 h following suspected AIS, using the stroke protocol. A modified Rankin scale (mRS) was used to evaluate the degree of functional dependence and disability three months after AIS. **Results**: The incidence of an unfavorable functional outcome evidently increased with more pronounced LA modalities (*p* < 0.05; χ^2^ test). The Kruskal–Wallis test found a statistically significant difference in aCBF values in relation to a degree of LA (*p* < 0.05). As there were a small number of multiple CMBs, no statistically significant difference was found based on the detection and degree of CMBs with aCBF and functional outcome; hence, the hypothesis was not entirely confirmed. **Conclusions**: This study indicates the reliability of MRI application in the initial diagnostic evaluation in order to gain an additional insight into the prediction of AIS outcomes. We demonstrated that LA correlates significantly with an unfavorable functional outcome after AIS, with decreased perfusion values. On the other hand, a higher proportion of unfavorable functional outcomes was observed in patients with CMBs. However, this result was not statistically significant and should be interpreted with caution.

## 1. Introduction

Acute ischemic stroke (AIS) is one of the leading causes of mortality worldwide and the primary cause of acquired neurological disability in adults, as well as dementia and epilepsy among elderly patients; thus it is an important medical and socio-economic issue [1,2,3]. Hence, following symptom recognition, the application of an adequate diagnostic procedure is required for the prompt selection of further treatment. Diagnostic accuracy in early AIS detection has been considerably improved by including novel advanced magnetic resonance imaging (MRI) techniques in clinical practice, thus enabling the prediction of outcomes [4]. The development of neuroradiology has introduced novel sequences, such as arterial spin labeling (ASL) and susceptibility-weighted angiography (SWAN), that, combined with standard techniques, demonstrate high sensitivity in the detection of ischemic tissue, the tissue at risk of ischemia, and the occlusion of a blood vessel, as well as thrombus localization and identification, and the detection of collaterals and microbleeds [5].

The fluid-attenuated inversion recovery (FLAIR) sequence is characterized by a strong T2 effect and the suppression of a signal originating from cerebrospinal fluid, and thus is highly sensitive in the detection of abnormal signals, primarily in the cortical region and periventricular area. As part of a standard magnetic resonance (MR) protocol in AIS patients, this technique plays multiple roles such as the detection of infarct age, a thrombosed blood vessel, and collaterals, but also the assessment of the degree of leukoaraiosis (LA) [6,7].

LA, also referred to as white matter lesions (WML), is described as a diffuse, confluent white matter abnormality of periventricular and subcortical localization, presented as hyperintensity on FLAIR [8].

The association between LA and neurological deterioration in AIS caused by a large blood vessel occlusion has been demonstrated, since LA is commonly regarded as a cerebral small vessel disease neuroimaging feature [9,10].

SWAN is an advanced gradient echo (GRE) sequence included in the MR protocol for ischemia. SWAN uses a long echo time (TE) to fully utilize the magnetic susceptibility effect, combined with a short TE that enables a more marked time-of-flight (TOF) effect [11].

This sequence provides significant predictive factors for a post-therapeutic outcome by allowing for the precise localization of a thrombus and insight into its structure, the detection of cerebral microbleeds (CMBs), and deoxyhemoglobin in the subependymal and deep medullary veins, i.e., “brush sign” [12,13,14].

CMBs consist of small, round, or ovoid perivascular hemosiderin deposits caused by a leakage in a small cerebral vessel [15], and are regarded as hemorrhagic and ischemic stroke markers that play a role in predicting post-stroke hemorrhagic complications [15,16].

A novel non-contrast perfusion technique, ASL, enables the detection of absolute cerebral blood flow (aCBF) values, thus allowing functional outcome prediction [5,17].

The present study seeks to examine the association of the degree of LA and CMBs with functional outcome prediction in patients with AIS. In addition, we aim to establish the correlation of the degree of LA and CMBs with aCBF values that would indirectly indicate the final AIS functional outcome. We hypothesize that the severity of LA and CMBs presented on an initial brain MR will be associated with lower aCBF values and an increased risk of an unfavorable functional outcome.

Most previous studies have evaluated LA and CMBs separately, without integrating ASL perfusion parameters in the AIS. The present study combines LA, CMBs, and ASL-derived aCBF values obtained within the first 24 h.

## 2. Materials and Methods

We conducted a cross-sectional study including a total of 205 consecutive male and female patients (aged ≥ 18 years) admitted urgently to Clinical Center Nis and Pitié Salpêtrière Hospital, Paris. All of the patients underwent brain MRI examinations in the first 24 h following suspected AIS. Patients admitted within 6 h of symptom onset were categorized as having hyperacute ischemic stroke (HAIS), whereas those admitted between 6 and 24 h were classified as AIS.

The inclusion criteria for patients’ participation were fulfilled by the clinical diagnostic criteria for AIS of the anterior cerebral circulation. The exclusion criteria were as follows: detected hemorrhagic stroke or acute ischemic stroke in the posterior cranial fossa, AIS imitators such as venous infarctions, PRES, seizure-related transient diffusion abnormalities, migraine-associated infarction-like changes, hypoglycemia-related neurological deficits, brain tumors, demyelinating lesions, other non-vascular conditions, AIS that was not confirmed by a neuroradiological method, artifacts resulting from a lack of patient cooperation that prevented the appropriate assessment of the obtained sequences, technical errors in examination performance, such as the incomplete acquisition of the required MRI sequences, severe artifacts compromising image quality, inappropriate perfusion labeling parameters in ASL acquisition not adjusted to patient age and other conditions, or failure of data reconstruction on the workstation, and absolute contraindications to MRI (e.g., metal implants or pacemakers). The study was approved by the Ethics Committees of both institutions.

We used the modified Rankin scale (mRS) to evaluate the degree of functional dependence and disability three months after AIS. This is a seven-point ordinal scale used to assess the level of disability or dependence in daily activities, ranging from no symptoms to severe disability that requires constant care and death outcome.

We applied a dichotomous distribution of patients: patients with favorable functional outcomes (mRS range 0–2), and patients with unfavorable functional outcomes (mRS range 3–5 and death 6).

The patients underwent radiological examination involving a brain MRI performed according to the protocol for ischemia.

All of the examinations were carried out on General Electric Healthcare (GE) SIGNA PIONEER (Florence, SC, USA) and HD23 MR scanners (Florence, SC, USA) (the field strength of 3 Tesla (T)) according to a 15 min ischemia protocol that included five sequences in the axial planes using standardized parameters such as diffusion-weighted imaging (DWI), ASL, SWAN, FLAIR and three-dimensional TOF angiography (3D TOF-MRA). We applied a fast three-dimensional pseudocontinuous (3D PCASL) ASL technique. 

A GE ADW 4.7 Workstation (Volume Viewer Version:13.0 Ext.2) was used to automatically generate the obtained data. No additional postprocessing was performed.

The region of interest (ROI) was placed on the ischemic lesion to determine the aCBF values on the ASL perfusion sequence.

The presence of LA was determined on the FLAIR sequence, by detecting periventricular and subcortical hyperintensity. The scoring of LA was performed via the Fazekas scale [18], designed for the assessment of the white matter hypersignal lesions. This scale evaluates periventricular and deep white matter hyperintensities separately. Periventricular hyperintensities are graded as follows: 0—absent; 1—“caps” or pencil-thin lining; 2—smooth “halo”; and 3—irregular hyperintensity extending into the deep white matter. Deep white matter hyperintensities are graded as follows: 0—absent; 1—punctate foci; 2—beginning confluence of foci; and 3—large confluent areas. For the statistical analyses, the overall LA burden was defined as the higher grade between periventricular and deep white matter hyperintensities, yielding a final score ranging from zero to three, as previously described by Srichawla et al. [19].

CMB areas were determined visually using the SWAN sequence. We looked for small (generally < 10 mm), round or ovoid hypointense lesions with a blooming effect on SWAN, distinct from vascular flow voids, calcifications, or artifacts. The CMBs were categorized according to their number as: no CMB, a few CMBs (less than ten), or multiple CMBs (including cases with more than ten lesions).

**Figure 1:**
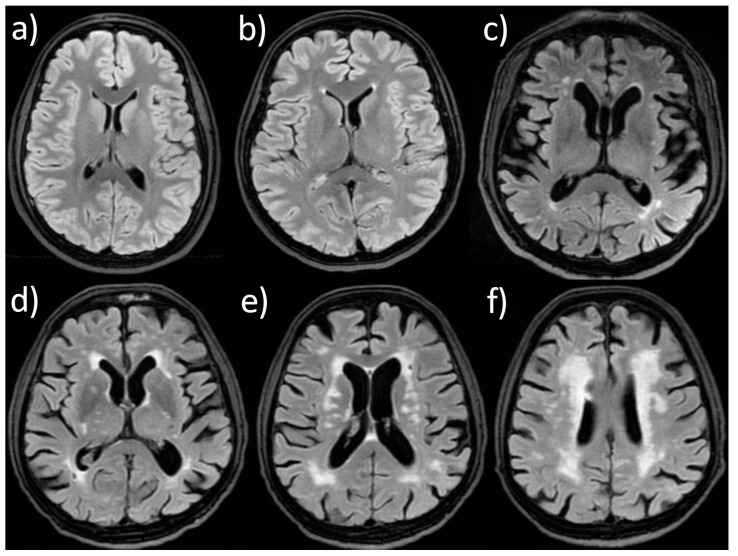
FLAIR images showing (**a**) the absence of LA; (**b**) caps on the frontal horns and a single punctate subcortical frontal focus—Fazekas 1; (**c**) caps around the occipital horns with several foci—Fazekas 1; (**d**) halo around the frontal and occipital horns—Fazekas 2; and (**e**,**f**) confluent periventricular and deep white matter LA in the same patient—Fazekas 3.

**Figure 2:**
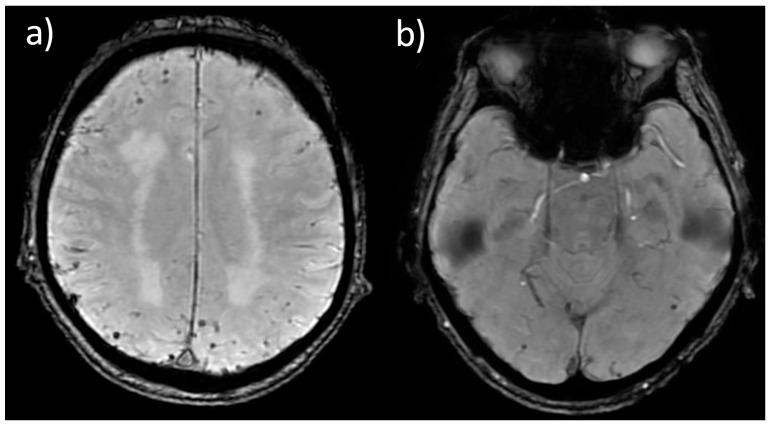
SWAN images showing (**a**) over ten CMBs bilaterally localized subcortically; (**b**) one lobar CMB in the right occipital lobe and one in the temporal lobe.

Representative examples of Fazekas grades of LA and the distribution of CMBs are presented in Figure 1 and Figure 2.

Statistical analyses were performed using SPSS, ver. 16.0. Continuous variables were expressed as the means ± SD (standard deviation) and Me (medians). The Shapiro–Wilk test was performed as a test of continuous variables normality. Categorical variables were expressed as frequencies—absolute numbers (N) and percentages (%). The chi-square test was used to assess differences between qualitative variables. The Mann–Whitney test was applied for continuous variables between two groups, and the Kruskal–Wallis test for comparisons among more than two groups. The statistical significance was defined as *p* < 0.05.

## 3. Results

The final cohort of 205 patients (108 (52.68%) males and 97 (47.32%) females; Table 1) was obtained after excluding 89 of the 294 patients with suspected AIS assessed in the Emergency Department. The exclusion criteria were noninterpretable ASL due to motion artifacts (*n* = 38), posterior fossa infarction (*n* = 24), the absence of infarction (*n* = 17), claustrophobia (*n* = 6), and stroke mimics (*n* = 4). There was no statistically significant difference in age, sex and the presence of tandem lesions between the groups of participants with hyperacute ischemic stroke (HAIS) within 6 h after stroke onset and AIS within 24 h after stroke onset. There was a statistically significant difference in frequencies of the left- and right-sided brain strokes between HAIS and AIS groups (*p* < 0.05) (Table 2). Table 3 shows the NIHSS values on admission and the aCBF values obtained by the ASL perfusion sequence in the entire sample. The obtained aCBF value was 29.62 ± 19.83 with the median value of 21.51, and there was no significant difference between the sexes (Table 4).

**Figure 3:**
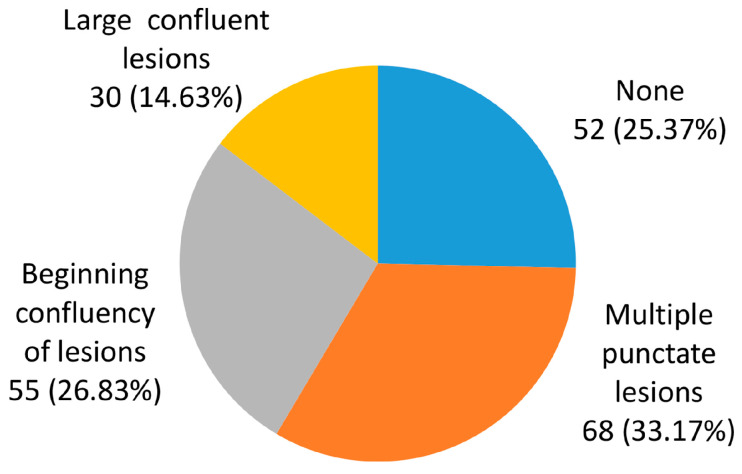
Patient distribution according to leukoaraiosis. 0—None; 1—multiple punctate lesions; 2—beginning confluency of lesions; and 3—large confluent lesions.

Figure 3 shows the distribution of LA degrees. The most prevalent modality was the presence of punctate lesions, observed in 68 (33.17%) of participants. LA was not observed in approximately a quarter (25.37%) of participants.

Table 5 shows AIS outcomes and the aCBF values in relation to LA degrees. The incidence of an unfavorable functional outcome evidently increased with more pronounced LA degrees (*p* < 0.05; χ^2^ test). There was a statistically significant higher incidence of favorable outcomes in the absence of LA (75.00% vs. 55.56%)—*p* < 0.05. The Kruskal–Wallis test found a statistically significant difference in the aCBF values in relation to the degree of LA, i.e., aCBF values were lower in the case of s more pronounced LA. The Mann–Whitney test found a statistically significant higher aCBF value in the absence of LA (*p* < 0.01).

**Figure 4:**
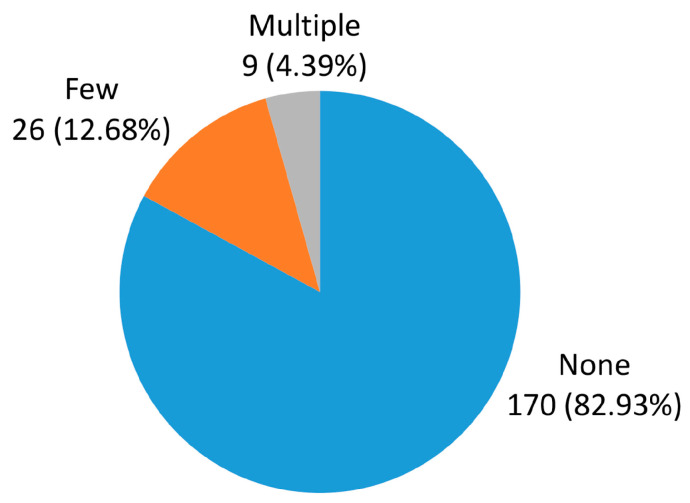
The distribution of microbleeds.

Figure 4 shows the presence of CMBs. Only 35 (17.07%) participants had CMBs, of which 9 (4.39%) participants had multiple CMBs.

As there was a small number of multiple CMBs, Table 6 shows the AIS outcomes and aCBF values in relation to the type and also the presence of CMBs. No statistically significant difference was found based on the data presented in the table, although the incidence of unfavorable outcomes was higher in participants with CMBs than in those without CMBs. Additionally, we found similar aCBF values in participants with and without CMBs, and in those with a small number of and multiple CMBs. Hence, the hypothesis that CMBs are related with an unfavorable outcome and lower aCBF values was not entirely confirmed. It should be noted that given the relatively small number of patients with multiple CMBs, the statistical power to detect significant differences may have been limited.

## 4. Discussion

**Figure 5:**
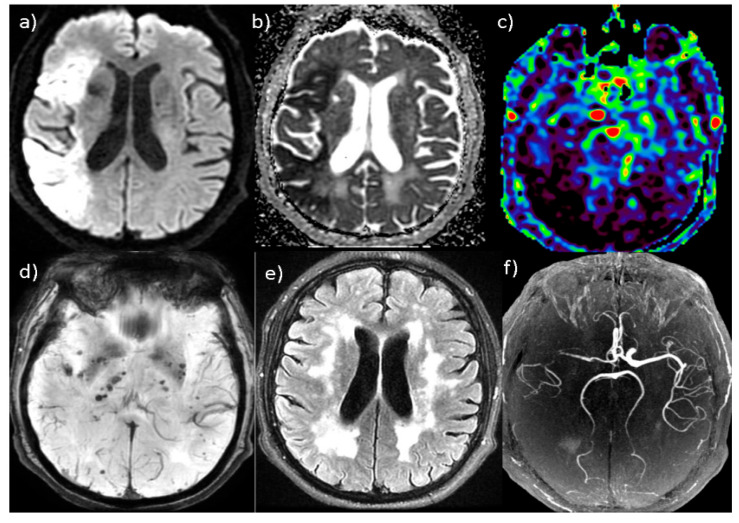
Brain MRI in hyperacute ischemic stroke patient: (**a**,**b**) axial DWI tomogram shows restricted diffusion in the irrigation of the right middle cerebral artery, with low ADC values; (**c**) 3D ASL perfusion revealed a hypoperfused right cerebral hemisphere; (**d**) axial SWAN tomogram detected multiple punctate cerebral microbleeds zones; (**e**) axial FLAIR tomogram shows large confluent white matter lesions; and (**f**) 3D TOF MRA shows occlusion of the right internal carotid and middle cerebral artery. MRI—magnetic resonance imaging; DWI—diffusion-weighted imaging; ADC—apparent diffusion coefficient; ASL—arterial spin labeling; SWAN—susceptibility-weighted angiography; FLAIR—fluid-attenuated inversion recovery; and TOF MRA—time-of-flight magnetic resonance angiography.

Predictors of clinical outcomes after stroke are required for the timely identification of high-risk patients and the application of adequate treatment. Advanced MRI techniques, such as SWAN and ASL sequences, are included in the imaging protocol for AIS and are of great importance for outcome prediction [20]. ASL is commercially obtainable and is applied routinely. Due to its short acquisition and interpretation time, this sequence plays an important role in obtaining high-quality diagnostic and prognostic data within a clinically relevant time that combined with findings on other sequences increase the accuracy of outcome prediction (Figure 5) [5].

On the FLAIR sequence, LA was detected in approximately three-quarters of the participants, with the greatest number (33.17%) presenting punctate lesions (Fazekas grade 1). We demonstrated a statistically significant difference in the incidence of a favorable and unfavorable functional outcome in the presence and absence of chronic microvascular WML (*p* < 0.05), whereby a favorable outcome statistically correlated with the absence of LA. In addition, we demonstrated a statistically significant correlation between aCBF values and the presence of LA (*p* < 0.05)—in the case of more pronounced LA, the aCBF values were lower.

The existing literature links a higher LA burden to poorer functional outcomes and AIS recurrence [21]. Additionally, as demonstrated in meta-analyses by Stewart et al., there is a clear association between LA severity and aCBF values [22]. These data suggest that the characteristics of lesions in small vessel disease are closely associated with reduced aCBF parameters. However, the overall relationship among LA burden, aCBF values, and functional results remains underexplored. Our study provides an integrated analysis correlating the LA burden, the aCBF values, and functional outcomes, offering insight into how chronic small vessel disease (as reflected by LA burden) can affect recovery and prognosis after an acute event. 

Most studies have reported the prevalence of LA as ranging from 40 to 80% [8,9,23]. Our results are in line with a study by Ren et al. [23] which found that patients with AIS had significantly unfavorable functional outcomes, especially with periventricular WML. In contrast to our work, a volumetric study by Griessenauer et al. [24] showed that while an increase in WML volume from 0 to 4 mL was associated with a decreased chance of a favorable functional outcome within 90 days, further increases above 4 mL were not statistically significant, and in patients with large vascular occlusions the WML volume was not an independent predictor of outcome. In a study by Zhang et al. [25], which included the measurement of aCBF using ASL, a significant negative relationship was found between aCBF and the volume of WML, indicating that a higher burden correlates with reduced cerebral perfusion. Our study is consistent with most previous research and indicates the importance of LA for the prediction of functional outcome after AIS. 

As part of the MR protocol for ischemia, the SWAN sequence allows for the detection of CMB zones that cannot be seen on conventional sequences. In our study CMBs were detected in 17.07% of participants, with multiple distributions found in only 4.39% of participants. 

The hypothesis that the incidence of an unfavorable functional outcome and lower aCBF values are related to the presence of CMBs was not entirely confirmed, most likely due to the infrequency of this modality among patients. The frequency of CMB in our sample was 17%, consistent with the previous literature reporting frequency ranges from 19% to 28% [26,27]. Nevertheless, this relatively low prevalence in the AIS population represents a limitation that reduces the statistical power of subgroup analyses, likely explaining why significant associations are often absent in smaller samples.

We demonstrated a higher incidence of unfavorable outcomes in patients with CMBs compared to those without CMBs. However, there was no statistically significant difference. In addition, we did not demonstrate lower aCBF values in participants with CMBs.

In addition, it is worth noting that direct evidence on the relationship between the aCBF values and CMB burden in AIS remains quite limited. By integrating structural small vessel disease markers with quantitative perfusion assessment, our study provides additional data on the interaction between chronic microvascular damage and acute hemodynamic impairment. In our research, we did not find a significant correlation between aCBF and CMB. This may suggest that acute perfusion impairment is more closely associated with large vessel pathology than with markers of small vessel disease. In line with the previous literature, some authors advise that isolated CMB presence should not automatically exclude patients from reperfusion therapy, particularly when no statistically significant association with functional outcome is observed [27]. Our findings further support a cautious and individualized interpretation of CMB burden in the acute setting. These findings provide additional insight into how structural changes and hemodynamic processes interact in the early phase of stroke.

In a study by Choi et al. [28], no statistically significant relationship was found between the presence of CMBs and a favorable outcome in the entire cohort of patients with AIS; on the contrary, in the recanalized subgroup, a high CMB burden and the lobar location of CMBs were independent predictors of poor 3-month outcome. Furthermore, a meta-analysis by Charidimou et al. [29] has demonstrated the association between the presence of CMBs and an increased risk for intracranial hemorrhage, and, consequently, an unfavorable functional outcome. The results of this analysis showed that the presence of more than five CMBs doubles the risk, while the presence of more than ten CMBs causes a fourfold increase in the risk for an unfavorable functional outcome. It should be noted, however, that these studies primarily focused on clinical and hemorrhagic outcomes and did not evaluate quantitative perfusion parameters such as aCBF.

Our study has several limitations. The small proportion of patients with CMBs limits our statistical power. As a result, we must interpret the lack of statistically significant connections cautiously, as the study may be underpowered to detect small-to-moderate CMB burden. In addition, the small number of patients with CMBs limited reliable subgroup analyses by anatomical location, including lobar and deep involvement.

In future studies, we aim to include a larger sample, facilitating detailed subgroup analyses not only of the LA degree and CMB burden and distribution, but also of additional clinical and imaging parameters relevant to AIS. Expanding the range of analyzed variables will enable a more detailed evaluation of prognosis and may help identify potential preventive strategies. Furthermore, the reporting of small vessel disease burden, especially LA, in routine radiological reports may improve risk assessment and support the use of preventive measures to reduce the risk of AIS.

## 5. Conclusions

This study indicates the reliability of MRI application in the initial diagnostic evaluation in order gaining an additional insight into the prediction of AIS outcome. We demonstrated that the presence and the degree of LA correlate significantly with an unfavorable functional outcome after AIS, with decreased perfusion values. On the other hand, a higher proportion of unfavorable functional outcomes was observed in patients with CMBs. However, this result was not statistically significant and should be interpreted with caution.

## Figures and Tables

**Table 1:** The demographic characteristics of patients.

Age		66.05 ± 15.59 (67.92)
Sex	Female	97 (47.32%)
	Male	108 (52.68%)

Age is expressed as the mean ± SD (median) and sex as frequencies and percentages.

**Table 2:** The demographic characteristics of HAIS and AIS patients.

		HAIS	AIS
Age		67.39 ± 15.48 (69.58)	65.31 ± 15.66 (67.01)
Sex	Female	34 (46.58%)	63 (47.73%)
	Male	39 (53.42%)	69 (52.27%)
Brain side *	Left	33 (45.21%)	79 (59.85%)
	Right	40 (54.79%)	53 (40.15%)
Tandem lesions		14 (19.18%)	15 (11.36%)

Continuous variables are expressed as X ± SD (Me), and categorical variables as frequencies and percentages. HAIS—hyperacute ischemic stroke; AIS—acute ischemic stroke, and *—*p* < 0.05 (chi-square test).

**Table 3:** The demographic characteristics of patients.

NIHSS admission	9.32 ± 7.90 (8.00)
aCBF value	29.62 ± 19.83 (21.51)

Variables are expressed as the mean ± SD (Me). aCBF—absolute cerebral blood flow.

**Table 4:** aCBF values regarding patients’ sex.

	Female	Male
aCBF	28.73 ± 18.15 (21.32)	30.42 ± 21.29 (21.83)

Variables are expressed as the mean ± SD (Me). aCBF—absolute cerebral blood flow.

**Table 5:** Functional outcome of ischemic stroke (mRS) and aCBF values in relation to the presence of leukoaraiosis.

	**Leukoaraiosis**			
	0 (N = 52)	1 (N = 68)	2 (N = 55)	3 (N = 30)
Outcome *				
Favorable	39 (75.00%) *	42 (61.76%)	27 (49.09%)	16 (53.33%)
Unfavorable	13 (25.00%)	26 (38.24%)	28 (50.91%)	14 (46.67%)
mRS (3–5)	11 (21.15%)	21 (30.88%)	25 (45.45%)	13 (43.33%)
Death	2 (3.85%)	5 (7.35%)	3 (5.45%)	1 (3.33%)
Outcome	Leukoaraiosis = No		Leukoaraiosis = Yes	
Favorable	39 (75.00%) *		85 (55.56%)	
Unfavorable	13 (25.00%)		68 (44.44%)	
	**Leukoaraiosis**			
	0	1	2	3
aCBF *	36.07 ± 23.50 (26.22)	30.21 ± 22.88 (21.17)	25.04 ± 13.12 (19.59)	25.50 ± 11.55 (20.48)
	Leukoaraiosis = No		Leukoaraiosis = Yes	
aCBF	36.07 ± 23.50 ** (26.22)		27.43 ± 17.99 (21.03)	

* *p* < 0.05 (the chi-square test; the Kruskal–Wallis test); ** *p* < 0.01 (the Mann–Whitney test). Continuous variables are expressed as the mean SD (Me), and categorical variables are expressed as frequencies and percentages. mRS—modified Rankin scale; aCBF—absolute cerebral blood flow; 0—none; 1—multiple punctate lesions; 2—beginning confluency of lesions; and 3—large confluent lesions.

**Table 6:** Functional outcome of ischemic stroke and aCBF values in correlation with the presence of microbleeds.

	**Microbleeds**			
	**None (n = 170)**	**Few (n = 26)**		**Multiple (n = 9)**
Outcome				
Favorable	106 (62.35%)	14 (53.85%)		4 (44.44%)
Unfavorable	64 (37.65%)	12 (46.15%)		5 (55.56%)
mRS (3–5)	55 (32.35%)	10 (38.46%)		5 (55.56%)
Death	9 (5.29%)	2 (7.69%)		0 (0.00%)
Outcome	Microbleeds = No		Microbleeds = Yes	
Favorable	106 (62.35%)		18 (51.43%)	
Unfavorable	64 (37.65%)		17 (48.57%)	
	**Microbleeds**			
	**None**	**Few**		**Multiple**
aCBF	29.68 ± 20.34 (21.50)	29.08 ± 18.42 (22.16)		30.12 ± 15.27 (24.50)
	Microbleeds = No		Microbleeds = Yes	
aCBF	29.68 ± 20.34 (21.50)		29.35 ± 17.45 (22.82)	

Continuous variables are expressed as X ± SD (Me), and categorical variables as frequencies and percentages. mRS—modified Rankin scale; aCBF—absolute cerebral blood flow.

## Data Availability

Data are available from the corresponding author upon reasonable request.

## Abbreviations

The following abbreviations are used in this manuscript:

AIS	Acute ischemic stroke
MRI	Magnetic resonance imaging
ASL	Arterial spin labeling
SWAN	Susceptibility-weighted angiography
FLAIR	Fluid-attenuated inversion recovery
T2	T2-weighted imaging
MR	Magnetic resonance
LA	Leukoaraiosis
WML	White matter lesions
GRE	Gradient echo
TE	Echo time
TOF	Time of flight
CMBs	Cerebral microbleeds
aCBF	Absolute cerebral blood flow
PRES	Posterior reversible encephalopathy syndrome
GE	General Electric Healthcare
DWI	Diffusion-weighted imaging
MRA	Magnetic resonance angiography
PCASL	Pseudocontinuous arterial spin labeling
ADW	Advantage workstation
ROI	Region of interest
SPSS	Statistical Package for the Social Sciences
mRS	Modified Rankin scale
HAIS	Hyperacute ischemic stroke
NIHSS	National Institutes of Health Stroke Scale
ADC	Apparent diffusion coefficient
